# Development of a microsurgery‐assisted robot for high‐precision thread traction and tension control, and confirmation of its applicability

**DOI:** 10.1002/rcs.2205

**Published:** 2020-12-01

**Authors:** Satoshi Hangai, Takahiro Nozaki, Tomoya Soma, Hidetaka Miyashita, Seiji Asoda, Masaki Yazawa, Kazuki Sato, Hiromasa Kawana, Kouhei Ohnishi, Eiji Kobayashi

**Affiliations:** ^1^ Department of System Design Engineering Keio University Minato Tokyo Japan; ^2^ Department of Mechanical Engineering Massachusetts Institute of Technology Cambridge Massachusetts USA; ^3^ Department of Dentistry and Oral Surgery Keio University Minato Tokyo Japan; ^4^ Department of Plastic and Reconstructive Surgery Keio University Minato Tokyo Japan; ^5^ Institute for Integrated Sports Medicine Keio University School of Medicine Shinjuku‐ku Tokyo Japan; ^6^ Department of Oral and Maxillofacial Implantology Kanagawa Dental University Yokosuka Kanagawa Japan; ^7^ Haptics Research Center Keio University Yokohama Japan; ^8^ Department of Organ Fabrication Keio University School of Medicine Shinjuku‐ku Tokyo Japan

**Keywords:** force control, haptics, lymphatic vessels, microsurgery, nerves, orthopaedic surgery, plastic surgery, robotics, surgery assistance, suture, traction, vessels

## Abstract

**Background:**

Microsurgery requires high skills for suturing using fragile threads, often within narrow surgical fields. Precise tension is required for good healing and to avoid the risk of thread breakage.

**Methods:**

To meet the demands, we developed a novel assist robot utilizing high‐precision sensorless haptic technology. The robot adopts a cable‐driven mechanism to maintain a distance from the surgical area and enhances compatibility with surgical equipment such as microscopes. The robot performance was verified through in vitro and in vivo experiments using a rat model.

**Results:**

The realization of precise tension control was confirmed in both experiments. In particular, in the in vivo experiments, the developed robot succeeded to produce a knot with an accurate tension of 0.66% error.

**Conclusions:**

The developed robot can realize to control traction force precisely. This technology might open up the window for a full assist robot for microsurgery with haptic feeling.

## 1 INTRODUCTION

Microsurgery is a procedure in which surgeons use a microscope to magnify the view of the surgical area, with sutures and anastomoses being performed on microscopic structures such as blood vessels, nerves, lymph ducts and so on. This surgical technology has been expanding its application range to maxillofacial surgery, head and neck surgery, gynaecology, ophthalmology, neurosurgery, plastic surgery and transplant surgery.^1^, ^2^, ^3^ Recently, robot‐assisted supermicrosurgery^4^ and the high‐performance video microscope^5^ have also been introduced.

Microsurgery is often performed in extremely narrow surgical fields, relying on visual information from microscopic images and using fragile suture threads. Therefore, the threads easily break if trainee surgeons apply a slightly excessive force. The thread fragility has been known to change depending on the suture diameter, knot type and suture material.^6^ Therefore, surgeons require a high level of expertise to skilfully perform these surgical procedures especially in traction operation and tension holding.

Robotic application has been considered a promising approach to assist surgical procedures and train surgeons.^7^, ^8^, ^9^ For macrosurgery, an important platform has been the Da Vinci Surgical System (Intuitive Surgical),^10^, ^11^, ^12^, ^13^ which is a teleoperation system comprising a master robot and a slave robot with two or three arms. Recently, microsurgical robotic technology has also been introduced wherein the surgeon manipulates the master robot, and the slave robot follows the motion accordingly in the surgical area.^4^, ^14^ This system has a great merit in assisting the performance of delicate tasks due to its features such as filtering the surgeon's tremor.

However, most surgical robots neglect force information. Due to the lack of force information, surgeons cannot adjust the robotic force applied to the target such as organs and sutures. Consequently, the surgery risks might be increased if inappropriate force is applied. In particular, thread traction during suturing operation in microsurgery becomes difficult in the absence of force information. Although it is also necessary to keep pulling the sutured thread when performing continuous suturing, continuing to maintain constant tension precisely and stably is very difficult. The fragile suture threads break if excessive force is applied, whereas a low force may result in a loose knot that unties after surgery. Tension accuracy is also closely related to post‐operative recovery.

Acquiring force information from the robots usually requires force or tactile sensors. Some surgical assist technologies have been developed using force sensors to assist delicate surgical procedures^15^, ^16^ or perform palpation.^17^ Although these sensors have been investigated and developed for these applications,^18^, ^19^, ^20^ force sensors are difficult to implement due to the limited space in the robot end effectors currently. In terms of the cost and benefits, these sensors also increase costs and degrade fault tolerance. Particularly in the case of precise force measurement, the sensor is fragile because it is necessary to use a sensor with a low allowable force in relation to dynamic range and resolution. Although some efforts to obtain force information without using force sensors have been reported, they have not yet achieved an accuracy and characteristics suitable for microsurgery. It is difficult to obtain accurate information on the low‐frequency component of the force using an accelerometer, and the method using fluid pressure causes a certain degree of error in transient conditions.^21^, ^22^, ^23^, ^24^

This paper describes the development of a microsurgery‐assisted robot that can precisely and stably control the tension in order to support traction and tension maintenance, which is one of the most advanced and skilful tasks. This system makes it possible for even a novice to perform the suturing process if he or she can make a loose knot. This system differs from conventional system with strain‐based force sensors in that it estimates the force from the electrical characteristics of the traction actuator and therefore does not break even if excessive force is applied.

## 2 MATERIALS AND METHODS

**FIGURE 1:**
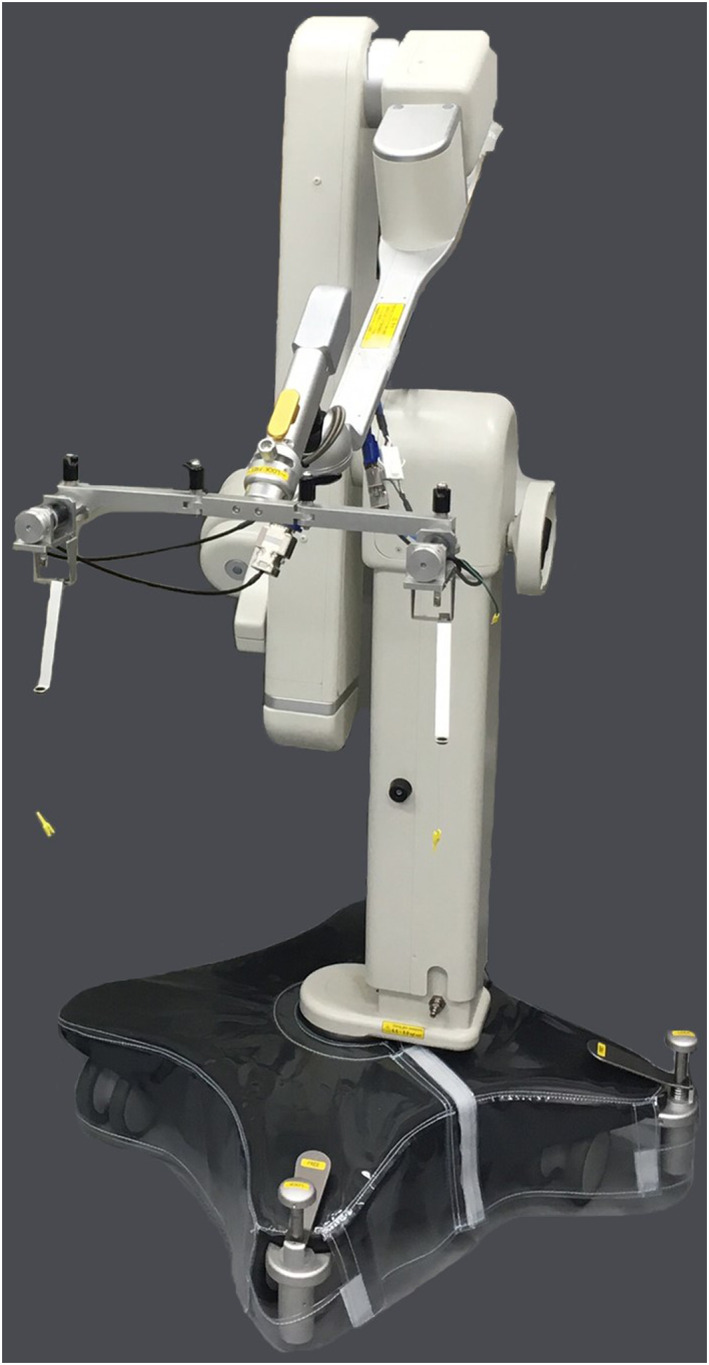
Developed microsurgery‐assist robot. The developed robot composed of the arm (Uni ARM; Mitaka Kohki) and winding parts

**FIGURE 2:**
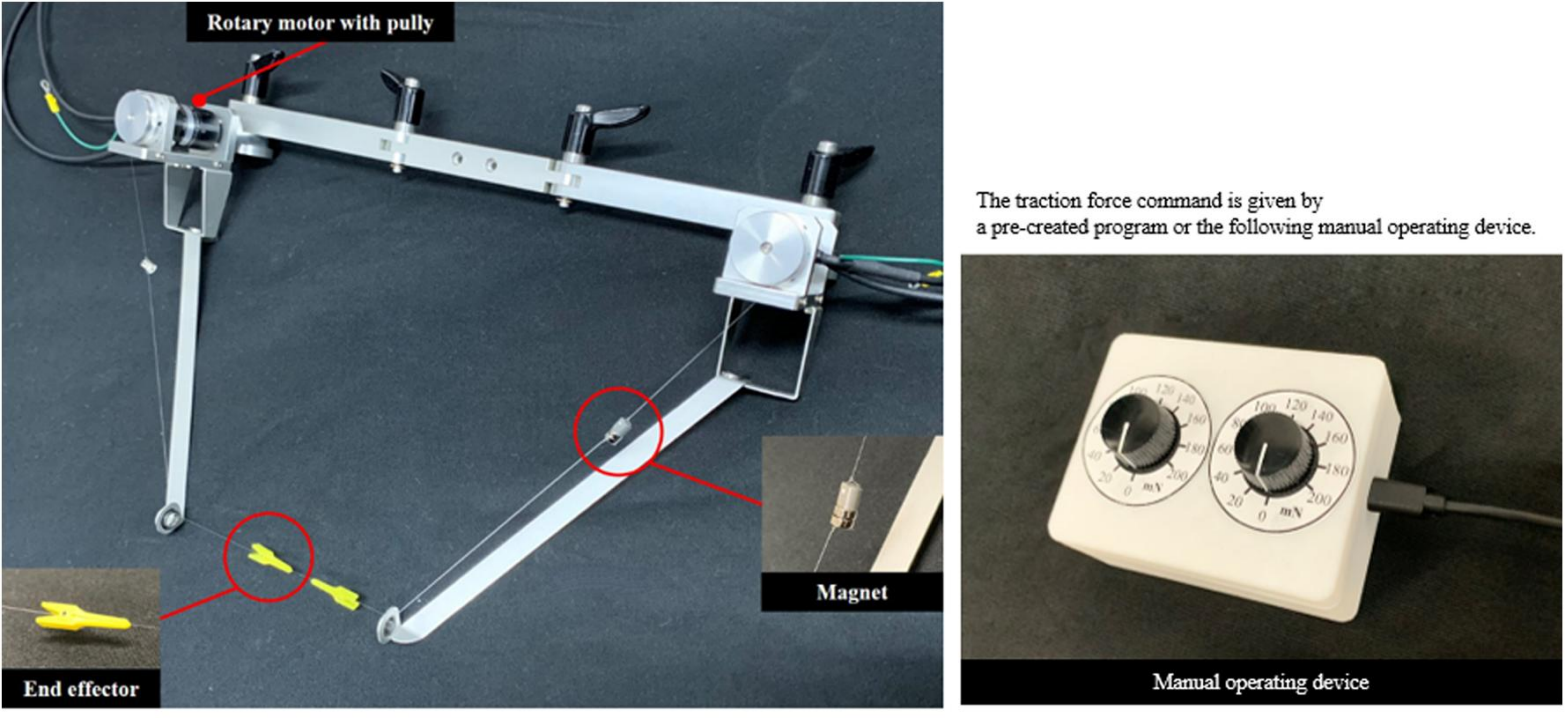
Detailed view of winding parts and manual operating device. Suture threads are held by the end effectors and wound by the rotary motors with nylon wire and magnets. The command of the traction force is given by a pre‐created program or manual operating device shown in this figure

**FIGURE 3:**
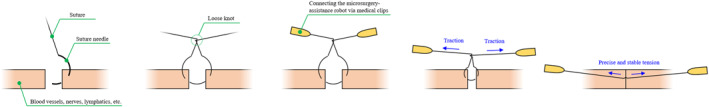
Procedure for using microsurgery‐assisted robot. The procedure of the microsurgery‐assisted robot and an overview of each process are shown

We then developed the microsurgery‐assisted robot by adhering to the above‐mentioned requirements. The developed robot winds the suture thread with fibres, by using a rotary motor. The overview of the robot is shown in Figure 1. The robot is mainly composed of an arm and the winding parts. The arm (Uni ARM, Mitaka Kohki) has a balancing mechanism and can easily fix its posture. A detailed view of the winding part is shown in Figure 2. The basic function of this robot is that when the surgeon activates the device, the winding part rotates and pulls the sutures to generate the appropriate tension through sensorless force control technology. The procedure for using the microsurgery‐assisted robot is shown in Figure 3. The surgeon will first manipulate the suture and the needle to create a loose knot on targeted tissues such as blood vessels, nerves and lymphatics. Note that this process is much easier than traction, although it is not performed by a robot. For precise and proper adjustment of the ligature force, the surgeon next holds both ends of a loosely tied suture with the tip to which the medical clip is connected (Disposable Micro Vascular Clip, Bear Medic). When the surgeon flips the switch, traction is initiated and the knot is tightened with precise and stable tension. The clips are sufficiently small to use in narrow surgical fields, thus providing adaptability. The end effectors are wound by rotary motors, using a nylon wire. By using a nylon wire for a power transfer mechanism, the robot can maintain a distance from the surgical field. Therefore, the compatibility requirement is satisfied. The middle section of the nylon wire is equipped with magnets as safety devices. If excessive tension is applied to the wire in case of failure, the magnets are disconnected and avoid winding the suture thread with high force. Therefore, safety is maintained even if the robot loses control. The magnet is connected to the microsurgery‐assisted robot or guide hole through the fibre, so it will not be lost if it is disconnected. In addition, the magnet will not fall into the body because the device is covered in practical use.

**FIGURE 4:**
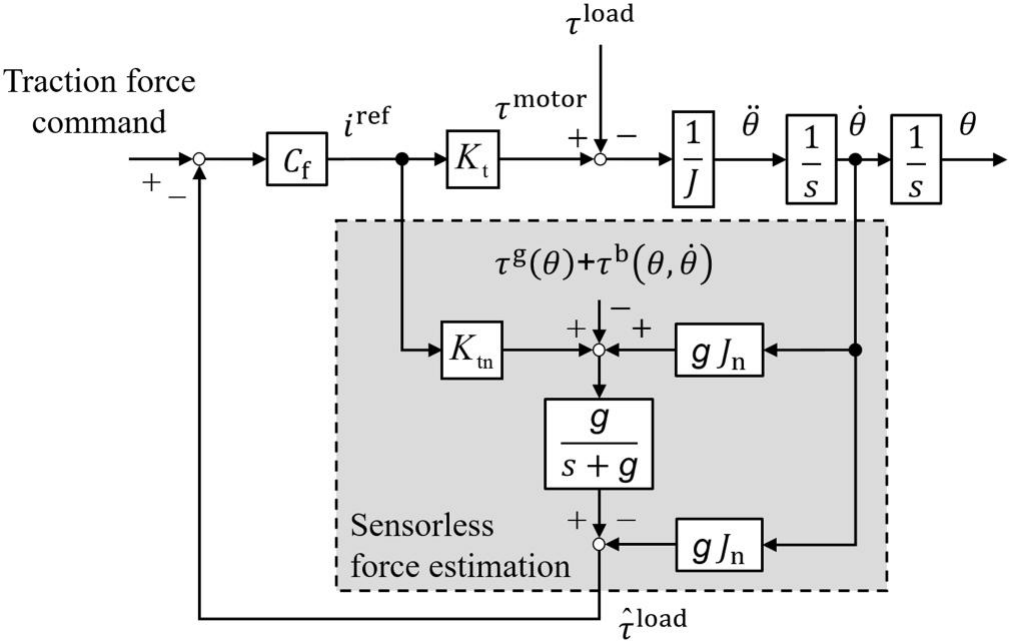
Block diagram of sensorless force measurement. The load force is estimated from the current reference and the angular velocity response

Figure 4 shows the block diagram of the force sensorless technology. The θ, J, $J_{\text{n}}$, $K_{\text{t}}$, $K_{\text{tn}}$, $C_{\text{f}}$, g, $\tau^{\text{load}}$, $\tau^{\text{motor}}$, $i^{\text{ref}}$ and s denote the angle, inertia, nominal inertia, torque constant, nominal torque constant, feedback controller, cut‐off frequency, load torque, motor torque, current reference and Laplace operator, respectively. The dots above the variables mean the derivatives and the hat means the estimated value. The basic principle of this force estimation is to estimate the disturbance force from the current reference and the angular velocity response, and then subtract the effects of gravity $\tau^{\text{g}}\left(\theta \right)$ and friction $\tau^{\text{b}}\left(\theta ,\;\dot{\theta }\right)$ from the estimated disturbance force to obtain the load force.

**FIGURE 5:**
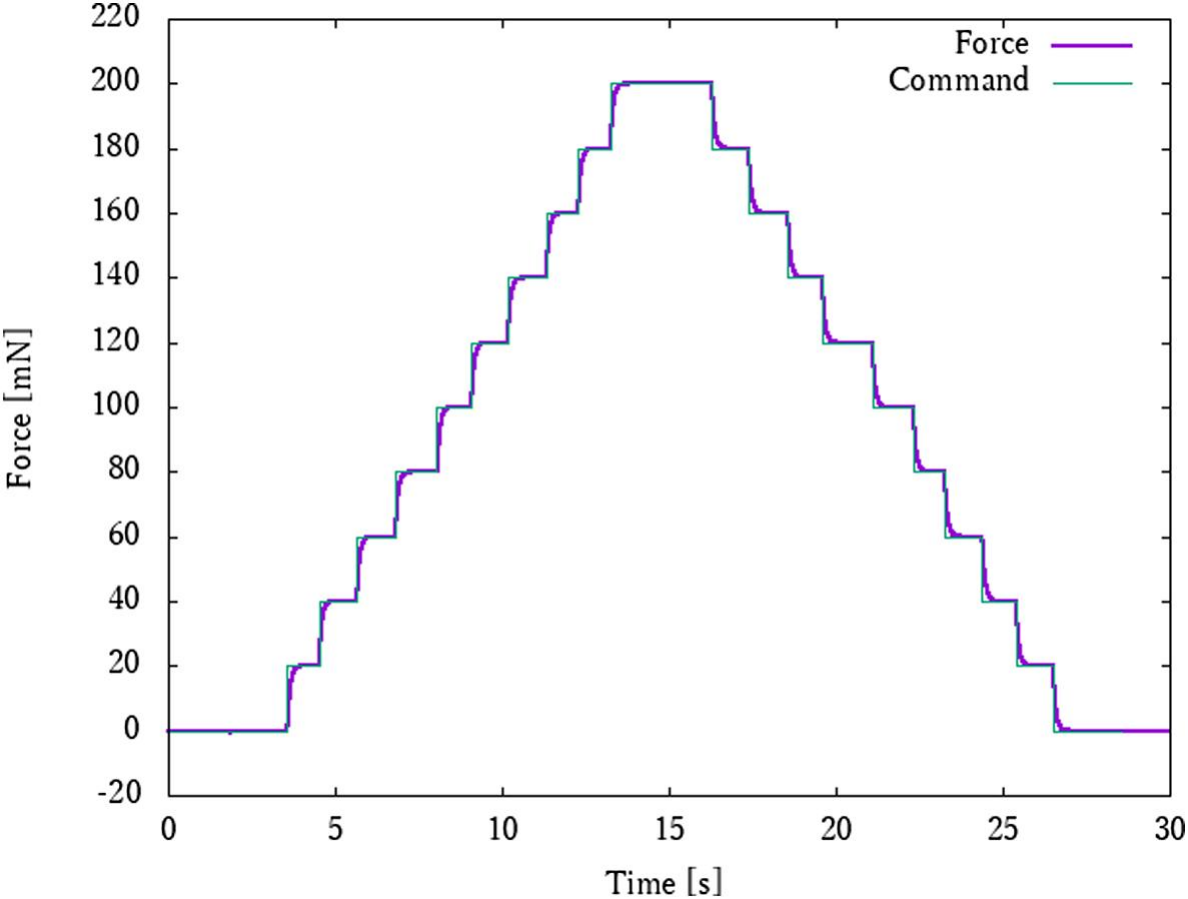
Result of accuracy verification test of developed microsurgery‐assist robot with sensor‐less force control system. The traction force was adjusted from 0 to 200 mN in this test. The result shows that there was very little error between the command and the response

The command value of the traction force can be given by a pre‐created program, or it can be adjusted manually by the operating device shown in Figure 2. In the latter case, by rotating the knob of the operating device, the traction force can be adjusted from 0 to 200 mN. Figure 5 shows the experimental results of the precise sensorless force control using the developed robot.

The range of possible winding force was determined by two types of in vitro experiments carried out beforehand.^1^

### 2.1 Rupture test (preliminary experiments for robot design)

The rupture test aimed to measure the characteristics of a suture, especially the force that causes the rupture, which was used to design the output range of the microsurgery‐assisted robot. Four types of sutures – 9‐0, 10‐0, 11‐0 and 12‐0 – were used in this mechanical study. Linear motors with the sensorless force estimation technology were used to apply traction to the sutures.^2^

### 2.2 Traction test (preliminary experiments for robot design)

The purpose of the traction test was to measure the actual force applied by a skilled surgeon to the thread during suturing. The measurement results were used to design the resolution and fast response of the forces produced by the microsurgery‐assisted robot. Four types of sutures – 9‐0, 10‐0, 11‐0 and 12‐0 – were also used in this study. One end of the suture was held by a skilled surgeon and the other end was connected to a linear motor controlled to a fixed position. Sensorless force estimation technology was implemented in this linear motor, and a skilled surgeon performed several traction actions in 10 s, at which time the traction force was measured.

**FIGURE 6:**
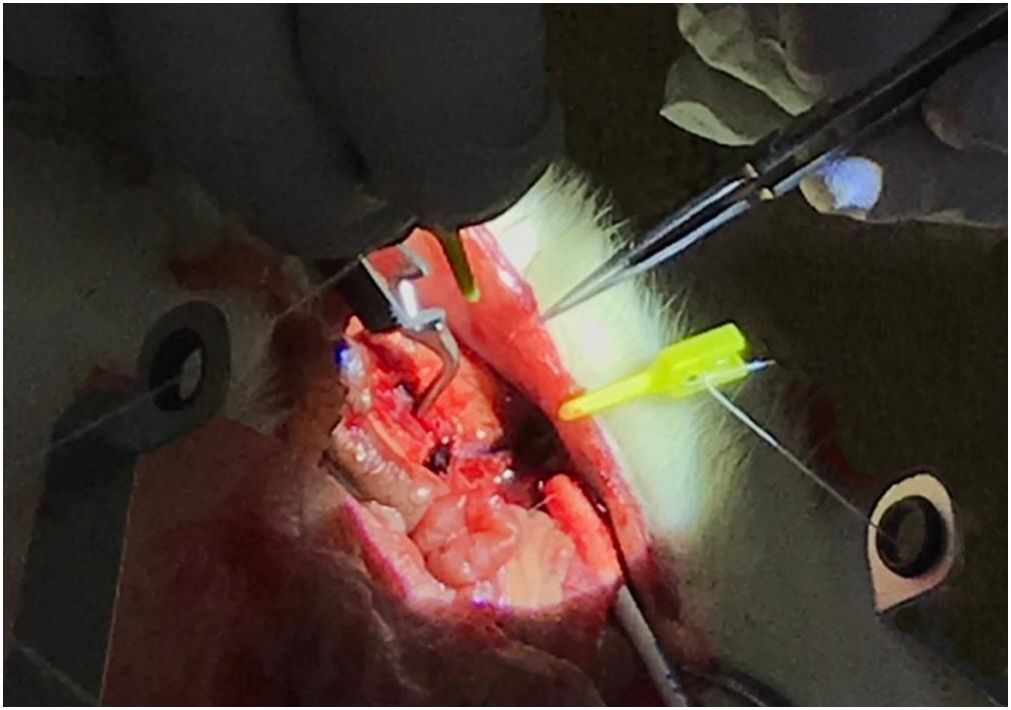
In vivo traction experiment to confirm its applicability to clinical practice. End effectors were used to hold the suture threads in narrow surgical fields

In the in vivo pre‐clinical study, a vascular anastomosis was performed using a rat model. Under sufficient inhalation anaesthesia, the rat was fixed in the supine position and a midline abdominal incision was made to open the abdomen. At the beginning of the test, the abdominal aorta and inferior vena cava of the rat were cut and their positions fixed. Then, a suture thread 9‐0 was inserted into the edge of the cutting surface and a loose knot was made. Both ends of the knot were held by the end effectors of the robot as shown in Figure 6. After this preparation, a surgeon set the traction force using the robot controller to complete the knotting.

The in vivo experimental protocols in this study were approved by the Institutional Animal Care and Use Committee of Keio University (Permit Number: 18011‐0), which operates in accordance with the Japanese Government for the care and use of laboratory animals. All surgical procedures were performed under general anaesthesia using medetomidine, midazolam and butorphanol tartrate. All efforts were made to minimize animal suffering. The rats were purchased from CLEA Japan. The animals were housed up to two rats per cage and kept under 12‐h light/dark cycles. Water and food were available ad libitum. The animals were euthanized by anaesthesia overdose 28 days after surgery.

We confirmed whether the traction force was appropriate after the completion of the knotting, by checking whether the knot was loose.

## 3 RESULTS

### 3.1 Mechanical tests for the development of the microsurgery‐assisted robot

**FIGURE 7:**
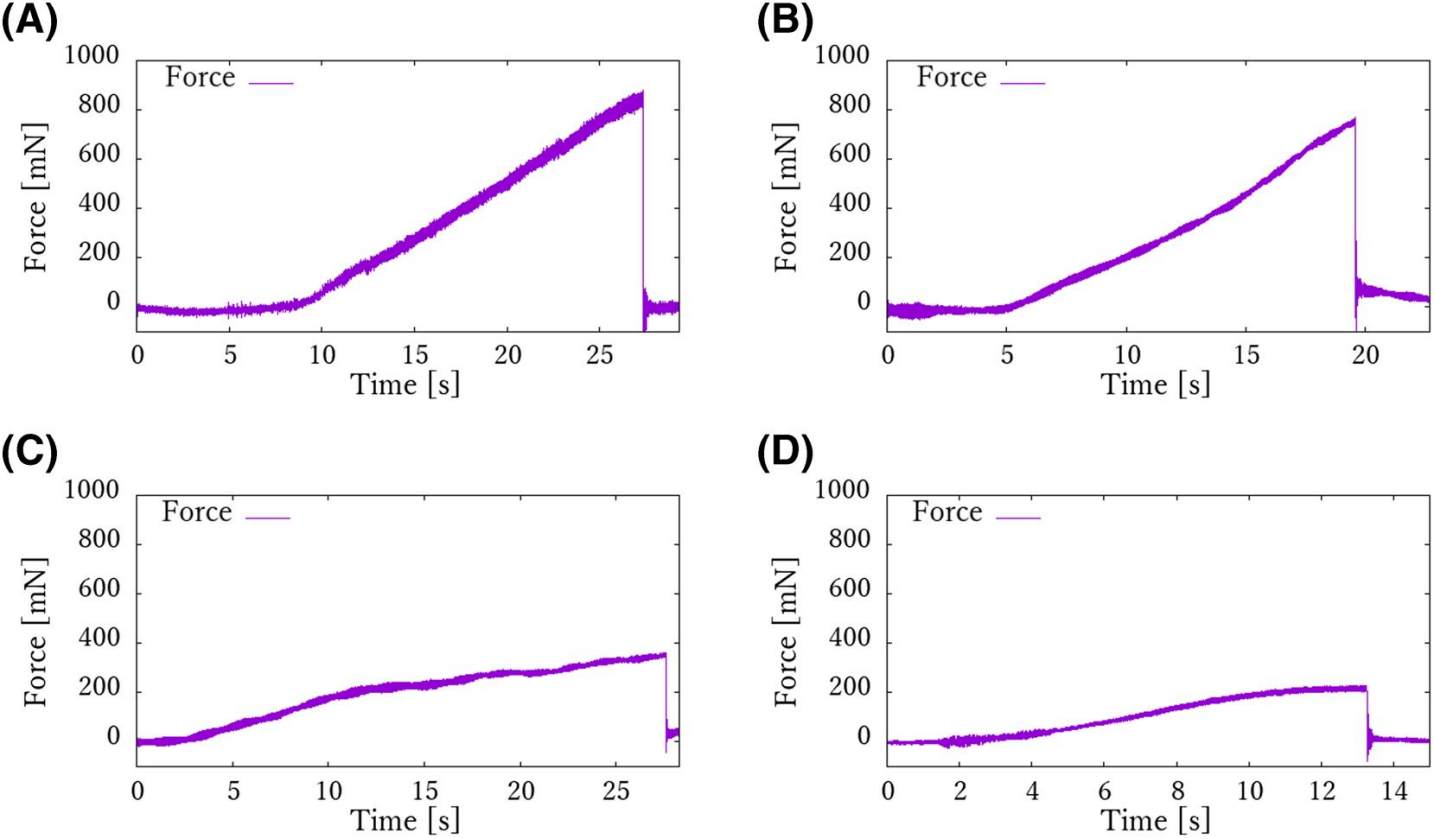
Results of rupture test. The traction force was gradually increased and the breaking force was measured. A response with a rapidly decreasing force indicates rupture. (A) Result of 9‐0 suture. (B) Result of 10‐0 suture. (C) Result of 11‐0 suture. (D) Result of 12‐0 suture

**FIGURE 8:**
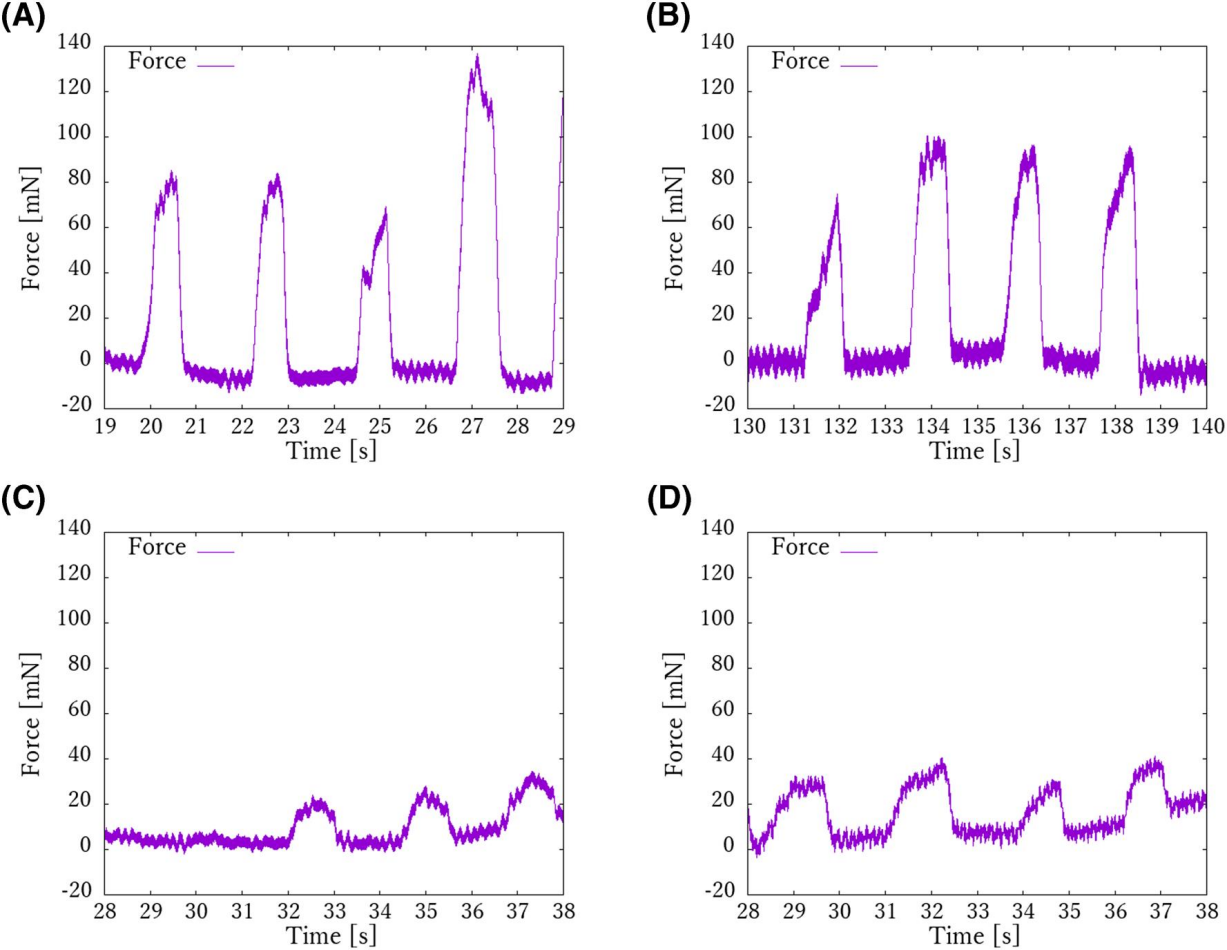
Results of traction test. How skilled surgeons apply tension force when pulling sutures was measured. (A) Result of 9‐0 suture. (B) Result of 10‐0 suture. (C) Result of 11‐0 suture. (D) Result of 12‐0 suture

In the rupture tests, the 9‐0, 10‐0, 11‐0 and 12‐0 sutures were ruptured by traction forces of 878.8, 767.4, 363.5 and 213.6 mN, respectively (Figure 7). On the other hand, the traction test results showed that the mean traction force of the 9‐0, 10‐0, 11‐0 and 12‐0 sutures was 93.8 ± 25.7, 91.9 ± 10.0, 28.9 ± 4.23 and 36.2 ± 4.50 mN, respectively (Figure 8). The traction force of the skilled surgeon in the traction test was confirmed to be sufficiently below the rupture force. Furthermore, for sutures from 9‐0 to 11‐0, the traction force of the skilled surgeon tended to decrease as the sutures became thinner, whereas the 12‐0 suture showed greater traction force than the 11‐0 suture. This result shows that it is particularly difficult to adjust the traction force for sutures thinner than 10‐0. In Figure 5, the light green line is the command given to the microsurgery‐assisted robot (input by a pre‐made program) and the purple line is the response of the force. The purple line shows the response value of the force. It can be confirmed that precise force control has been successfully achieved.

### 3.2 In vivo experiment using the developed microsurgery‐assisted robot

**FIGURE 9:**
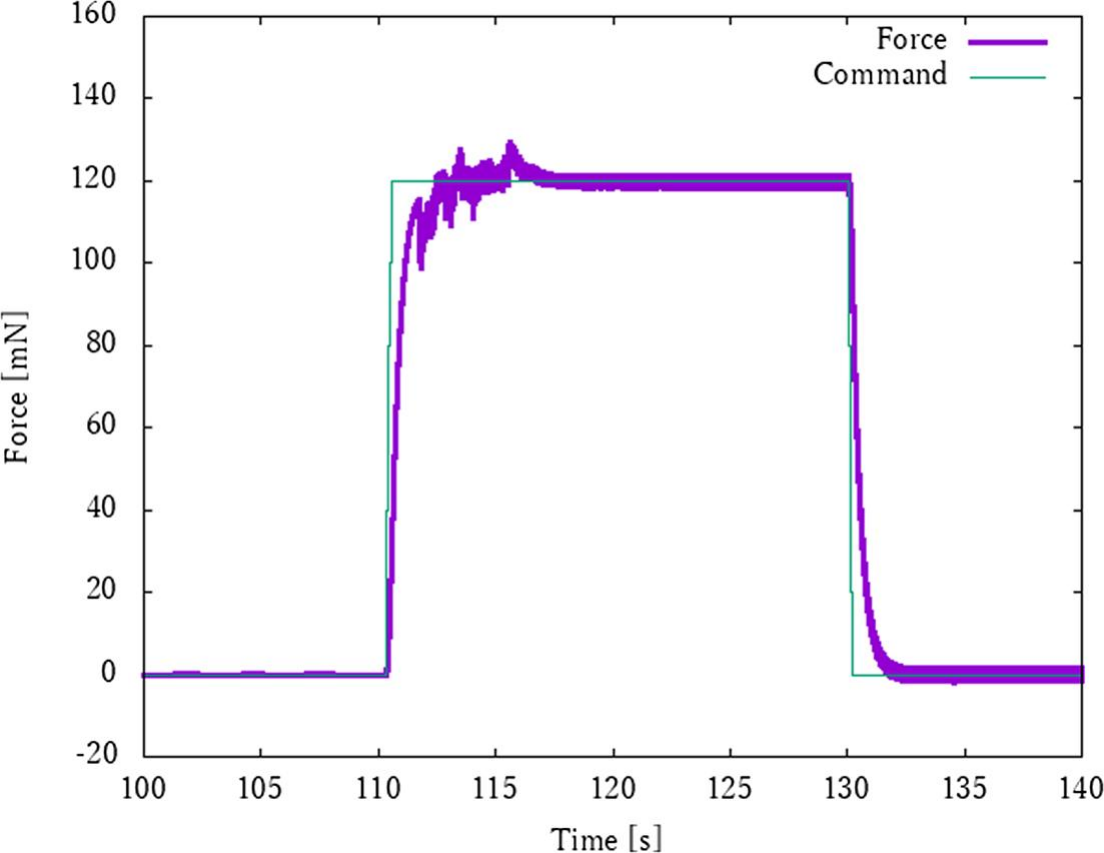
Step response of traction force in in vivo experiment. The developed robot succeeded to apply 120 mN tension force precisely, with an error between the force response and command in steady state (117–130 s) of only 0.66%

**FIGURE 10:**
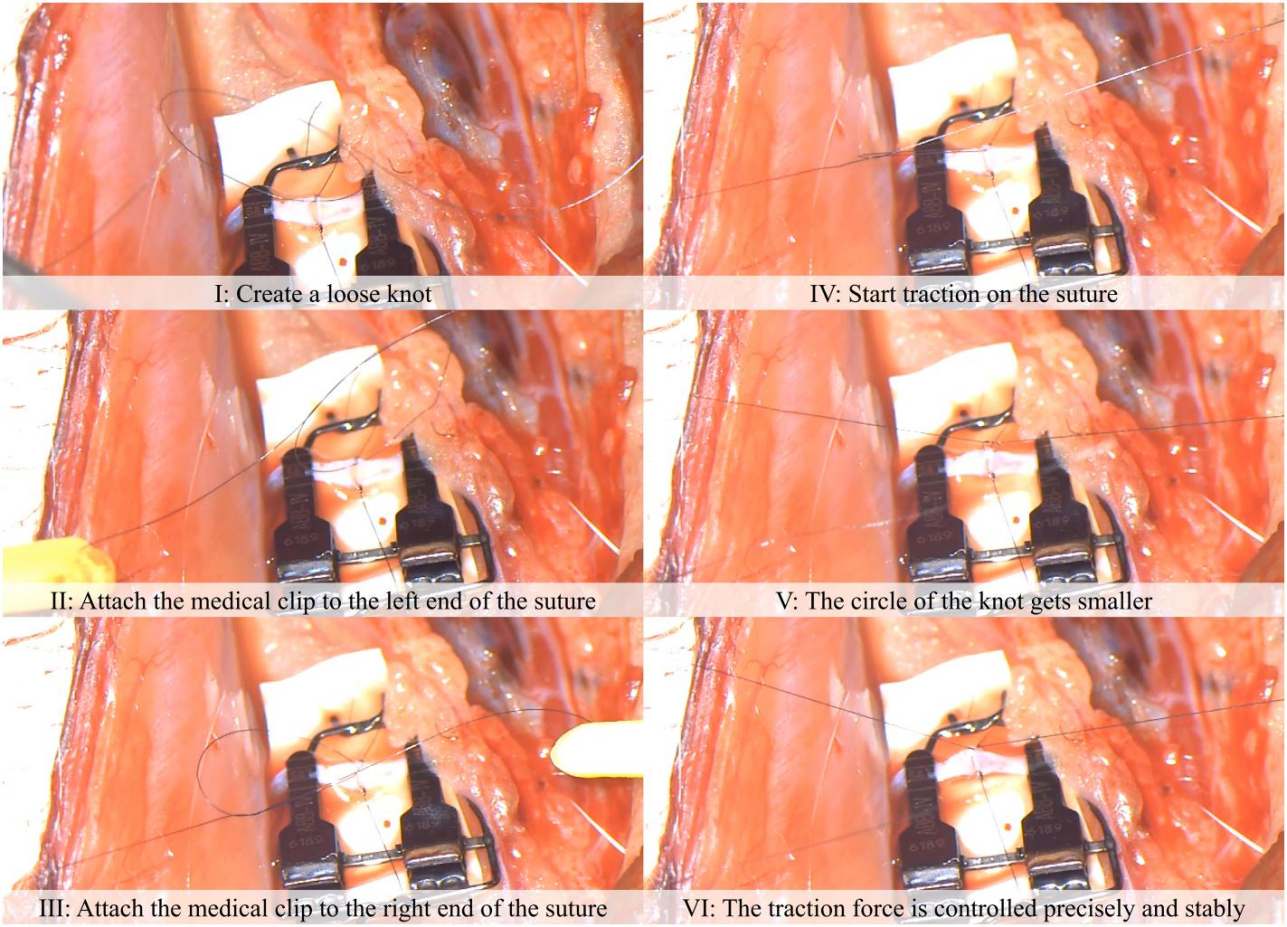
Sequence of steps to complete tighten knot. The knot‐tightening process is completed by securing the clip in a loose knot and switching on the robot

The resulting force response is shown in Figure 9. As shown in the figure, the robot traction force was controlled precisely and stably to the specified value, obtaining an error between the force response and command in steady state (117–130 s) of only 0.66%. Owing to this preciseness, a tight knot was successfully prepared without breaking the suture thread. The sequence of steps to tighten the knot and the images are shown in Figure 10. These experimental results show that this system can achieve one of the most difficult tasks, that is, pulling and maintaining suture tension, and this system can greatly assist the surgeon in microsurgery. In all trials of the in vivo experiments, it was confirmed that the sutures were 100% safe, with no breakage or loosening after suturing.

## 4 DISCUSSION

We developed a microsurgery‐assisted robot to realize delicate suture knotting with high precision, in place of a skilled surgeon. The developed robot performs knotting by winding the suture thread with rotary motors. The suture thread is held with small clips and wound with a nylon wire. Therefore, the robot maintains a distance from the surgical field and is compatible with the existing medical equipment. To control the winding force precisely, sensorless force control is implemented. This reduces the cost of the robot and enhances its fault tolerance. The developed robot realizes accurate force control. The effectiveness of the developed robot was verified through an in vivo experiment. The robot succeeded in knotting a suture with precise force control, obtaining a control error (difference between the command and the response) of only 0.66% (i.e., 0.795 mN).

Until now, haptic sensation has been used in very few conventional robots.^10^, ^11^, ^12^, ^13^ Although attempts have been made to mount haptics on robots in the past,^15^, ^16^, ^17^ most of them have been large and costly with sensors mounted on the tips.

To develop our robot, we first measured the traction used by experts in microsurgery and then built a prototype to feed that traction in the robot. Based on the data from experiments, the main range of the traction force for the assistant robot was set from 0 to 200 mN.

The ultimate goal of this study is to complete difficult knots that require delicate and precise knot force adjustments on behalf of a skilled surgeon. In order to get there, this paper reported the development and results of a microsurgery‐assisted robot that can precisely and stably control the tension in order to support traction and tension maintenance, which is one of the most advanced and skilful tasks. As shown in the experimental results, the developed robot realizes precise traction force control for ensuring that the thread does not break and the knot stays firmly in place. Force information can be obtained from observers implemented in the control algorithms.^25^, ^26^, ^27^ In this method, force information is estimated based on the position, thus enabling sensorless force control. If a typical commercial motor driver is used, calibration is not necessary because the information needed to estimate the force is the current reference calculated by the controller and the measured position value. The accuracy of the force control in the in vivo experiment was sufficiently high, to reproduce the delicate tasks as performed by skilled surgeons.

Some robotic teleoperation systems, such as the Da Vinci Surgical System,^10^, ^11^, ^12^, ^13^ have been reported to assist surgical tasks by realizing telesurgery or filtering the surgeons' tremor. However, most conventional teleoperation systems do not utilize force information, which increases the risk of medical accidents due to the difficulty of force adjustment. By applying the observer‐based force estimation to the robotic teleoperation system, it can be expected that many delicate and complicated tasks, such as suturing, become easier without increasing costs or degrading fault tolerance.

Although the fixation arm, strings and clips used in this study are commercially available for medical use, it is necessary to select and install motors and covers that are appropriate for medical robots before the entire device can be sold on the market. The device falls under Class II of medical devices; and therefore, the performance and reliability of the device itself was confirmed first through the experiments described in this paper. We anticipate further work verifying the clinical prognosis of the device from a clinical perspective.

## 5 CONCLUSIONS

We developed a microsurgery‐assisted robot to realize the delicate traction control with high precision for support difficult operation of the microsurgery. In vitro and in vivo experiments were conducted to confirm the performance of the device and its applicability to actual suture traction work. The results revealed the breaking force of the microfine thread and the waveform of the tension applied by a skilled surgeon. In addition, the very fine traction forces that make microsurgery difficult were successfully controlled. The accuracy of the traction force control is very high compared to other approaches using force sensors to acquire force information. The comparison with the movement information of a skilled surgeon confirmed that the traction force control performance of this robot far exceeds that of a skilled surgeon. Since this device enabled precise control of tension, it is expected to clarify the relationship between tension and knot quality and post‐operative recovery in the future. It is also hoped that, in the future, the applicability of this system will be expanded and this system will serve as an alternative to skilled surgeons.

## CONFLICTS OF INTEREST

Seiji Asoda received the funding from Japan Agency for Medical Research and Development (AMED). The funder had no role in study design, data collection and analysis, decision to publish, or preparation of the manuscript.
